# Supplementary material for: Expression-linked promoter selection (ELiPS) engineers short, strong ubiquitous promoters for gene therapy applications
Source: bioRxiv. 2026 Jun 26:2026.06.25.734611. Preprint. [Version 1] doi: 10.64898/2026.06.25.734611 (PMC13320777; doi:10.64898/2026.06.25.734611)
Supplement: Supplement 2 [file media-2.pdf]

Supplementary Table 1: Identity and description of transcription factors, binding sites, and barcodes used in ELiPS libraries 1 and 2

Library 1	TF Name	Ensembl	Gene Description	TFBS Sequence	Barcode	Orientation	Top oligo BbsI	Bottom oligo BbsI	Top oligo BsaI	Bottom oligo BsaI
	ATF4	ENSG00000128272	Activating transcription factor 4	GGATGATGCAAT	GTCT	Forward	caaaGGATGATGCAATcaaagaGTCTTCaaaGAAGACacactcGTCT	gagtAGACgagtgtGTCTTCtttGAAGACtctttgATTGCATCATCC	caaaGGATGATGCAATcaaagGAGACCaaaGGTCTCcactcGTCT	gagtAGACgagtgGAGACCtttGGTCTCctttgATTGCATCATCC
	FOS	ENSG00000170345	Fos proto-oncogene, AP-1 transcription factor subunit	TGTGACTCAT	CTAC	Forward	caaaTGTGACTCATcaaagaGTCTTCaaaGAAGACacactcCTAC	gagtGTAGgagtgtGTCTTCtttGAAGACtctttgATGAGTCACA	caaaTGTGACTCATcaaagGAGACCaaaGGTCTCcactcCTAC	gagtGTAGgagtgGAGACCtttGGTCTCctttgATGAGTCACA
	JUN	ENSG00000177606	Jun proto-oncogene, AP-1 transcription factor subunit	AAGATGATGTCAT	AGAA	Forward	caaaAAGATGATGTCATcaaagaGTCTTCaaaGAAGACacactcAGAA	gagtTTCTgagtgtGTCTTCtttGAAGACtctttgATGACATCATCTT	caaaAAGATGATGTCATcaaagGAGACCaaaGGTCTCcactcAGAA	gagtTTCTgagtgGAGACCtttGGTCTCctttgATGACATCATCTT
	CEBPB	ENSG00000172216	CCAAT/enhancer binding protein beta	TATTGCACAAT	CCCA	Forward	caaaTATTGCACAATcaaagaGTCTTCaaaGAAGACacactcCCCA	gagtTGGGgagtgtGTCTTCtttGAAGACtctttgATTGTGCAATA	caaaTATTGCACAATcaaagGAGACCaaaGGTCTCcactcCCCA	gagtTGGGgagtgGAGACCtttGGTCTCctttgATTGTGCAATA
	NFYA	ENSG00000001167	Nuclear transcription factor Y subunit alpha	CTCAGCCAATCAGCG	TAAG	Forward	caaaCTCAGCCAATCAGCGcaaagaGTCTTCaaaGAAGACacactcTAAG	gagtCTTAgagtgtGTCTTCtttGAAGACtctttgCGCTGATTGGCTGAG	caaaCTCAGCCAATCAGCGcaaagGAGACCaaaGGTCTCcactcTAAG	gagtCTTAgagtgGAGACCtttGGTCTCctttgCGCTGATTGGCTGAG
	EGR1	ENSG00000120738	Early growth response 1	CCCCCGCCCCCGCC	TCGG	Forward	caaaCCCCCGCCCCCGCCcaaagaGTCTTCaaaGAAGACacactcTCGG	gagtCCGAgagtgtGTCTTCtttGAAGACtctttgGGCGGGGGCGGGGG	caaaCCCCCGCCCCCGCCcaaagGAGACCaaaGGTCTCcactcTCGG	gagtCCGAgagtgGAGACCtttGGTCTCctttgGGCGGGGGCGGGGG
	NFE2L2	ENSG00000116044	Nuclear factor, erythroid 2 like 2	ATGACTCAGCA	ACCC	Forward	caaaATGACTCAGCAcaaagaGTCTTCaaaGAAGACacactcACCC	gagtGGGTgagtgtGTCTTCtttGAAGACtctttgTGCTGAGTCAT	caaaATGACTCAGCAcaaagGAGACCaaaGGTCTCcactcACCC	gagtGGGTgagtgGAGACCtttGGTCTCctttgTGCTGAGTCAT
	NR1H2	ENSG00000131408	Nuclear receptor subfamily 1 group H member 2	AAAGGTCAAAGGTCAAC	CCAA	Forward	caaaAAAGGTCAAAGGTCAACcaaagaGTCTTCaaaGAAGACacactcCCAA	gagtTTGGgagtgtGTCTTCtttGAAGACtctttgGTTGACCTTTGACCTTT	caaaAAAGGTCAAAGGTCAACcaaagGAGACCaaaGGTCTCcactcCCAA	gagtTTGGgagtgGAGACCtttGGTCTCctttgGTTGACCTTTGACCTTT
	CREB1	ENSG00000118260	CAMP responsive element binding protein 1	TGACGTCA	TCGT	Forward	caaaTGACGTCAcaaagaGTCTTCaaaGAAGACacactcTCGT	gagtACGAgagtgtGTCTTCtttGAAGACtctttgTGACGTCA	caaaTGACGTCAcaaagGAGACCaaaGGTCTCcactcTCGT	gagtACGAgagtgGAGACCtttGGTCTCctttgTGACGTCA
	KLF6	ENSG00000067082	Kruppel like factor 6	GCCACGCCCA	AAGG	Forward	caaaGCCACGCCCAcaaagaGTCTTCaaaGAAGACacactcAAGG	gagtCCTTgagtgtGTCTTCtttGAAGACtctttgTGGGCGTGGC	caaaGCCACGCCCAcaaagGAGACCaaaGGTCTCcactcAAGG	gagtCCTTgagtgGAGACCtttGGTCTCctttgTGGGCGTGGC
	ETS2	ENSG00000157557	ETS proto-oncogene 2, transcription factor	GACCGGAAGT	GGAT	Forward	caaaGACCGGAAGTcaaagaGTCTTCaaaGAAGACacactcGGAT	gagtATCCgagtgtGTCTTCtttGAAGACtctttgACTTCCGGTC	caaaGACCGGAAGTcaaagGAGACCaaaGGTCTCcactcGGAT	gagtATCCgagtgGAGACCtttGGTCTCctttgACTTCCGGTC
	ELF1	ENSG00000120690	E74 like ETS transcription factor 1	GAACCAGGAAGTG	CCTG	Forward	caaaGAACCAGGAAGTGcaaagaGTCTTCaaaGAAGACacactcCCTG	gagtCAGGgagtgtGTCTTCtttGAAGACtctttgCACTTCCTGGTTC	caaaGAACCAGGAAGTGcaaagGAGACCaaaGGTCTCcactcCCTG	gagtCAGGgagtgGAGACCtttGGTCTCctttgCACTTCCTGGTTC
	TCF12	ENSG00000140262	Transcription factor 12	CACGTGC	CACG	Forward	caaaCACGTGCcaaagaGTCTTCaaaGAAGACacactcCACG	gagtCGTGgagtgtGTCTTCtttGAAGACtctttgGCACGTG	caaaCACGTGCcaaagGAGACCaaaGGTCTCcactcCACG	gagtCGTGgagtgGAGACCtttGGTCTCctttgGCACGTG
	SP1	ENSG00000185591	Sp1 transcription factor	GGGGCGGGGT	GTAT	Forward	caaaGGGGCGGGGTcaaagaGTCTTCaaaGAAGACacactcGTAT	gagtATACgagtgtGTCTTCtttGAAGACtctttgACCCCGCCCC	caaaGGGGCGGGGTcaaagGAGACCaaaGGTCTCcactcGTAT	gagtATACgagtgGAGACCtttGGTCTCctttgACCCCGCCCC
	POU2F1	ENSG00000143190	POU class 2 homeobox 1	AATATGCAAATAA	GAGA	Forward	caaaAATATGCAAATAAcaaagaGTCTTCaaaGAAGACacactcGAGA	gagtTCTCgagtgtGTCTTCtttGAAGACtctttgTTATTTGCATATT	caaaAATATGCAAATAAcaaagGAGACCaaaGGTCTCcactcGAGA	gagtTCTCgagtgGAGACCtttGGTCTCctttgTTATTTGCATATT
	ATF4_rev	ENSG00000128272	Activating transcription factor 4	ATTGCATCATCC	AGCC	Reverse	caaaATTGCATCATCCcaaagaGTCTTCaaaGAAGACacactcAGCC	gagtGGCTgagtgtGTCTTCtttGAAGACtctttgGGATGATGCAAT	caaaATTGCATCATCCcaaagGAGACCaaaGGTCTCcactcAGCC	gagtGGCTgagtgGAGACCtttGGTCTCctttgGGATGATGCAAT
	FOS_rev	ENSG00000170345	Fos proto-oncogene, AP-1 transcription factor subunit	ATGAGTCACA	TGTG	Reverse	caaaATGAGTCACAcaaagaGTCTTCaaaGAAGACacactcTGTG	gagtCACAgagtgtGTCTTCtttGAAGACtctttgTGTGACTCAT	caaaATGAGTCACAcaaagGAGACCaaaGGTCTCcactcTGTG	gagtCACAgagtgGAGACCtttGGTCTCctttgTGTGACTCAT
	JUN_rev	ENSG00000177606	Jun proto-oncogene, AP-1 transcription factor subunit	ATGACATCATCTT	TTAT	Reverse	caaaATGACATCATCTTcaaagaGTCTTCaaaGAAGACacactcTTAT	gagtATAAgagtgtGTCTTCtttGAAGACtctttgAAGATGATGTCAT	caaaATGACATCATCTTcaaagGAGACCaaaGGTCTCcactcTTAT	gagtATAAgagtgGAGACCtttGGTCTCctttgAAGATGATGTCAT
	CEBPB_rev	ENSG00000172216	CCAAT/enhancer binding protein beta	ATTGTGCAATA	TACG	Reverse	caaaATTGTGCAATAcaaagaGTCTTCaaaGAAGACacactcTACG	gagtCGTAgagtgtGTCTTCtttGAAGACtctttgTATTGCACAAT	caaaATTGTGCAATAcaaagGAGACCaaaGGTCTCcactcTACG	gagtCGTAgagtgGAGACCtttGGTCTCctttgTATTGCACAAT
	NFYA_rev	ENSG00000001167	Nuclear transcription factor Y subunit alpha	CGCTGATTGGCTGAG	CAGA	Reverse	caaaCGCTGATTGGCTGAGcaaagaGTCTTCaaaGAAGACacactcCAGA	gagtTCTGgagtgtGTCTTCtttGAAGACtctttgCTCAGCCAATCAGCG	caaaCGCTGATTGGCTGAGcaaagGAGACCaaaGGTCTCcactcCAGA	gagtTCTGgagtgGAGACCtttGGTCTCctttgCTCAGCCAATCAGCG
	EGR1_rev	ENSG00000120738	Early growth response 1	GGCGGGGGCGGGGG	CGAA	Reverse	caaaGGCGGGGGCGGGGGcaaagaGTCTTCaaaGAAGACacactcCGAA	gagtTTCGgagtgtGTCTTCtttGAAGACtctttgCCCCCGCCCCCGCC	caaaGGCGGGGGCGGGGGcaaagGAGACCaaaGGTCTCcactcCGAA	gagtTTCGgagtgGAGACCtttGGTCTCctttgCCCCCGCCCCCGCC
	NFE2L2_rev	ENSG00000116044	Nuclear factor, erythroid 2 like 2	TGCTGAGTCAT	ATTC	Reverse	caaaTGCTGAGTCATcaaagaGTCTTCaaaGAAGACacactcATTC	gagtGAATgagtgtGTCTTCtttGAAGACtctttgATGACTCAGCA	caaaTGCTGAGTCATcaaagGAGACCaaaGGTCTCcactcATTC	gagtGAATgagtgGAGACCtttGGTCTCctttgATGACTCAGCA
	NR1H2_rev	ENSG00000131408	Nuclear receptor subfamily 1 group H member 2	GTTGACCTTTGACCTTT	ACAA	Reverse	caaaGTTGACCTTTGACCTTTcaaagaGTCTTCaaaGAAGACacactcACAA	gagtTTGTgagtgtGTCTTCtttGAAGACtctttgAAAGGTCAAAGGTCAAC	caaaGTTGACCTTTGACCTTTcaaagGAGACCaaaGGTCTCcactcACAA	gagtTTGTgagtgGAGACCtttGGTCTCctttgAAAGGTCAAAGGTCAAC
	CREB1_rev	ENSG00000118260	CAMP responsive element binding protein 1	TGACGTCA	GTCC	Reverse	caaaTGACGTCAcaaagaGTCTTCaaaGAAGACacactcGTCC	gagtGGACgagtgtGTCTTCtttGAAGACtctttgTGACGTCA	caaaTGACGTCAcaaagGAGACCaaaGGTCTCcactcGTCC	gagtGGACgagtgGAGACCtttGGTCTCctttgTGACGTCA
	KLF6_rev	ENSG00000067082	Kruppel like factor 6	TGGGCGTGGC	AGGC	Reverse	caaaTGGGCGTGGCcaaagaGTCTTCaaaGAAGACacactcAGGC	gagtGCCTgagtgtGTCTTCtttGAAGACtctttgGCCACGCCCA	caaaTGGGCGTGGCcaaagGAGACCaaaGGTCTCcactcAGGC	gagtGCCTgagtgGAGACCtttGGTCTCctttgGCCACGCCCA
	ETS2_rev	ENSG00000157557	ETS proto-oncogene 2, transcription factor	ACTTCCGGTC	TCCT	Reverse	caaaACTTCCGGTCcaaagaGTCTTCaaaGAAGACacactcTCCT	gagtAGGAgagtgtGTCTTCtttGAAGACtctttgGACCGGAAGT	caaaACTTCCGGTCcaaagGAGACCaaaGGTCTCcactcTCCT	gagtAGGAgagtgGAGACCtttGGTCTCctttgGACCGGAAGT
	ELF1_rev	ENSG00000120690	E74 like ETS transcription factor 1	CACTTCCTGGTTC	CCCT	Reverse	caaaCACTTCCTGGTTCcaaagaGTCTTCaaaGAAGACacactcCCCT	gagtAGGGgagtgtGTCTTCtttGAAGACtctttgGAACCAGGAAGTG	caaaCACTTCCTGGTTCcaaagGAGACCaaaGGTCTCcactcCCCT	gagtAGGGgagtgGAGACCtttGGTCTCctttgGAACCAGGAAGTG
	TCF12_rev	ENSG00000140262	Transcription factor 12	GCACGTG	GATA	Reverse	caaaGCACGTGcaaagaGTCTTCaaaGAAGACacactcGATA	gagtTATCgagtgtGTCTTCtttGAAGACtctttgCACGTGC	caaaGCACGTGcaaagGAGACCaaaGGTCTCcactcGATA	gagtTATCgagtgGAGACCtttGGTCTCctttgCACGTGC
	SP1_rev	ENSG00000185591	Sp1 transcription factor	ACCCCGCCCC	GTTG	Reverse	caaaACCCCGCCCCcaaagaGTCTTCaaaGAAGACacactcGTTG	gagtCAACgagtgtGTCTTCtttGAAGACtctttgGGGGCGGGGT	caaaACCCCGCCCCcaaagGAGACCaaaGGTCTCcactcGTTG	gagtCAACgagtgGAGACCtttGGTCTCctttgGGGGCGGGGT
	POU2F1_rev	ENSG00000143190	POU class 2 homeobox 1	TTATTTGCATATT	CGGA	Reverse	caaaTTATTTGCATATTcaaagaGTCTTCaaaGAAGACacactcCGGA	gagtTCCGgagtgtGTCTTCtttGAAGACtctttgAATATGCAAATAA	caaaTTATTTGCATATTcaaagGAGACCaaaGGTCTCcactcCGGA	gagtTCCGgagtgGAGACCtttGGTCTCctttgAATATGCAAATAA

Library 2	TF Name	Ensembl	Gene Description	TFBS Sequence	Barcode	Orientation	Top oligo BbsI	Bottom oligo BbsI	Top oligo BsaI	Bottom oligo BsaI
	HNF1A	ENSG00000135100	HNF1 homeobox A	AGTTAATGATTAACT	TGCG	Forward	caaaAGTTAATGATTAACTcaaagaGTCTTCaaaGAAGACacactcTGCG	gagtCGCAgagtgtGTCTTCtttGAAGACtctttgAGTTAATCATTAACT	caaaAGTTAATGATTAACTcaaagGAGACCaaaGGTCTCcactcTGCG	gagtCGCAgagtgGAGACCtttGGTCTCctttgAGTTAATCATTAACT
	HNF1B	ENSG00000275410	HNF1 homeobox B	GTTAATGATTAAC	TGAA	Forward	caaaGTTAATGATTAACcaaagaGTCTTCaaaGAAGACacactcTGAA	gagtTTCAgagtgtGTCTTCtttGAAGACtctttgGTTAATCATTAAC	caaaGTTAATGATTAACcaaagGAGACCaaaGGTCTCcactcTGAA	gagtTTCAgagtgGAGACCtttGGTCTCctttgGTTAATCATTAAC
	HNF4A	ENSG00000101076	hepatocyte nuclear factor 4, alpha	CTGGACTTTGGCCTC	GGCG	Forward	caaaCTGGACTTTGGCCTCcaaagaGTCTTCaaaGAAGACacactcGGCG	gagtCGCCgagtgtGTCTTCtttGAAGACtctttgGAGGCCAAAGTCCAG	caaaCTGGACTTTGGCCTCcaaagGAGACCaaaGGTCTCcactcGGCG	gagtCGCCgagtgGAGACCtttGGTCTCctttgGAGGCCAAAGTCCAG
	NR2F1	ENSG00000175745	nuclear receptor subfamily 2, group F, member 1	CAAAGGTCAAGGA	ACGG	Forward	caaaCAAAGGTCAAGGAcaaagaGTCTTCaaaGAAGACacactcACGG	gagtCCGTgagtgtGTCTTCtttGAAGACtctttgTCCTTGACCTTTG	caaaCAAAGGTCAAGGAcaaagGAGACCaaaGGTCTCcactcACGG	gagtCCGTgagtgGAGACCtttGGTCTCctttgTCCTTGACCTTTG
	TBP	ENSG00000112592	TATA box binding protein	GTATAAAAGGCGGGG	GCGC	Forward	caaaGTATAAAAGGCGGGGcaaagaGTCTTCaaaGAAGACacactcGCGC	gagtGCGCgagtgtGTCTTCtttGAAGACtctttgCCCCGCCTTTTATAC	caaaGTATAAAAGGCGGGGcaaagGAGACCaaaGGTCTCcactcGCGC	gagtGCGCgagtgGAGACCtttGGTCTCctttgCCCCGCCTTTTATAC
	FOXA2	ENSG00000125798	forkhead box A2	TGTTTACTTAGG	CGAC	Forward	caaaTGTTTACTTAGGcaaagaGTCTTCaaaGAAGACacactcCGAC	gagtGTCGgagtgtGTCTTCtttGAAGACtctttgCCTAAGTAAACA	caaaTGTTTACTTAGGcaaagGAGACCaaaGGTCTCcactcCGAC	gagtGTCGgagtgGAGACCtttGGTCTCctttgCCTAAGTAAACA
	FOXD1	ENSG00000251493	forkhead box D1	GTAAACAT	TAAA	Forward	caaaGTAAACATcaaagaGTCTTCaaaGAAGACacactcTAAA	gagtTTTAgagtgtGTCTTCtttGAAGACtctttgATGTTTAC	caaaGTAAACATcaaagGAGACCaaaGGTCTCcactcTAAA	gagtTTTAgagtgGAGACCtttGGTCTCctttgATGTTTAC
	FOXF2	ENSG00000137273	forkhead box F2	CAAACGTAAACAAT	AGTA	Forward	caaaCAAACGTAAACAATcaaagaGTCTTCaaaGAAGACacactcAGTA	gagtTACTgagtgtGTCTTCtttGAAGACtctttgATTGTTTACGTTTG	caaaCAAACGTAAACAATcaaagGAGACCaaaGGTCTCcactcAGTA	gagtTACTgagtgGAGACCtttGGTCTCctttgATTGTTTACGTTTG
	FOXA1	ENSG00000129514	forkhead box A1	TCCATGTTTACTTTG	GTTC	Forward	caaaTCCATGTTTACTTTGcaaagaGTCTTCaaaGAAGACacactcGTTC	gagtGAACgagtgtGTCTTCtttGAAGACtctttgCAAAGTAAACATGGA	caaaTCCATGTTTACTTTGcaaagGAGACCaaaGGTCTCcactcGTTC	gagtGAACgagtgGAGACCtttGGTCTCctttgCAAAGTAAACATGGA
	PLAG1	ENSG00000181690	pleiomorphic adenoma gene 1	GGGGCCCAAGGGGG	TCTA	Forward	caaaGGGGCCCAAGGGGGcaaagaGTCTTCaaaGAAGACacactcTCTA	gagtTAGAgagtgtGTCTTCtttGAAGACtctttgCCCCCTTGGGCCCC	caaaGGGGCCCAAGGGGGcaaagGAGACCaaaGGTCTCcactcTCTA	gagtTAGAgagtgGAGACCtttGGTCTCctttgCCCCCTTGGGCCCC
	GABPA	ENSG00000154727	GA binding protein transcription factor, alpha subunit 60kDa	CCGGAAGTGGC	CTAA	Forward	caaaCCGGAAGTGGCcaaagaGTCTTCaaaGAAGACacactcCTAA	gagtTTAGgagtgtGTCTTCtttGAAGACtctttgGCCACTTCCGG	caaaCCGGAAGTGGCcaaagGAGACCaaaGGTCTCcactcCTAA	gagtTTAGgagtgGAGACCtttGGTCTCctttgGCCACTTCCGG
	MYB	ENSG00000118513	v-myb myeloblastosis viral oncogene homolog (avian)	ACCAACTGTC	CGAG	Forward	caaaACCAACTGTCcaaagaGTCTTCaaaGAAGACacactcCGAG	gagtCTCGgagtgtGTCTTCtttGAAGACtctttgGACAGTTGGT	caaaACCAACTGTCcaaagGAGACCaaaGGTCTCcactcCGAG	gagtCTCGgagtgGAGACCtttGGTCTCctttgGACAGTTGGT
	NFE2L2	ENSG00000116044	nuclear factor (erythroid-derived 2)-like 2	ATGACTCAGCA	TGGC	Forward	caaaATGACTCAGCAcaaagaGTCTTCaaaGAAGACacactcTGGC	gagtGCCAgagtgtGTCTTCtttGAAGACtctttgTGCTGAGTCAT	caaaATGACTCAGCAcaaagGAGACCaaaGGTCTCcactcTGGC	gagtGCCAgagtgGAGACCtttGGTCTCctttgTGCTGAGTCAT
	CREB1	ENSG00000118260	cAMP responsive element binding protein 1	GGTGACGTCACC	GTTT	Forward	caaaGGTGACGTCACCcaaagaGTCTTCaaaGAAGACacactcGTTT	gagtAAACgagtgtGTCTTCtttGAAGACtctttgGGTGACGTCACC	caaaGGTGACGTCACCcaaagGAGACCaaaGGTCTCcactcGTTT	gagtAAACgagtgGAGACCtttGGTCTCctttgGGTGACGTCACC
	EGR1	ENSG00000120738	Early Growth Response 1	TACGCCCACGCATT	TGGT	Forward	caaaTACGCCCACGCATTcaaagaGTCTTCaaaGAAGACacactcTGGT	gagtACCAgagtgtGTCTTCtttGAAGACtctttgAATGCGTGGGCGTA	caaaTACGCCCACGCATTcaaagGAGACCaaaGGTCTCcactcTGGT	gagtACCAgagtgGAGACCtttGGTCTCctttgAATGCGTGGGCGTA
	E2F1	ENSG00000101412	E2F1	TTTGGCGCCAAA	AGAT	Forward	caaaTTTGGCGCCAAAcaaagaGTCTTCaaaGAAGACacactcAGAT	gagtATCTgagtgtGTCTTCtttGAAGACtctttgTTTGGCGCCAAA	caaaTTTGGCGCCAAAcaaagGAGACCaaaGGTCTCcactcAGAT	gagtATCTgagtgGAGACCtttGGTCTCctttgTTTGGCGCCAAA
	NRF1	ENSG00000106459	Nuclear respiratory factor 1	TGCGCACGCGCA	GGGA	Forward	caaaTGCGCACGCGCAcaaagaGTCTTCaaaGAAGACacactcGGGA	gagtTCCCgagtgtGTCTTCtttGAAGACtctttgTGCGCGTGCGCA	caaaTGCGCACGCGCAcaaagGAGACCaaaGGTCTCcactcGGGA	gagtTCCCgagtgGAGACCtttGGTCTCctttgTGCGCGTGCGCA
	HNF1A_rev	ENSG00000135100	HNF1 homeobox A	AGTTAATCATTAACT	CTAG	Reverse	caaaAGTTAATCATTAACTcaaagaGTCTTCaaaGAAGACacactcCTAG	gagtCTAGgagtgtGTCTTCtttGAAGACtctttgAGTTAATGATTAACT	caaaAGTTAATCATTAACTcaaagGAGACCaaaGGTCTCcactcCTAG	gagtCTAGgagtgGAGACCtttGGTCTCctttgAGTTAATGATTAACT
	HNF1B_rev	ENSG00000275410	HNF1 homeobox B	GTTAATCATTAAC	TACA	Reverse	caaaGTTAATCATTAACcaaagaGTCTTCaaaGAAGACacactcTACA	gagtTGTAgagtgtGTCTTCtttGAAGACtctttgGTTAATGATTAAC	caaaGTTAATCATTAACcaaagGAGACCaaaGGTCTCcactcTACA	gagtTGTAgagtgGAGACCtttGGTCTCctttgGTTAATGATTAAC
	HNF4A_rev	ENSG00000101076	hepatocyte nuclear factor 4, alpha	GAGGCCAAAGTCCAG	GATT	Reverse	caaaGAGGCCAAAGTCCAGcaaagaGTCTTCaaaGAAGACacactcGATT	gagtAATCgagtgtGTCTTCtttGAAGACtctttgCTGGACTTTGGCCTC	caaaGAGGCCAAAGTCCAGcaaagGAGACCaaaGGTCTCcactcGATT	gagtAATCgagtgGAGACCtttGGTCTCctttgCTGGACTTTGGCCTC
	NR2F1_rev	ENSG00000175745	nuclear receptor subfamily 2, group F, member 1	TCCTTGACCTTTG	GAAA	Reverse	caaaTCCTTGACCTTTGcaaagaGTCTTCaaaGAAGACacactcGAAA	gagtTTTCgagtgtGTCTTCtttGAAGACtctttgCAAAGGTCAAGGA	caaaTCCTTGACCTTTGcaaagGAGACCaaaGGTCTCcactcGAAA	gagtTTTCgagtgGAGACCtttGGTCTCctttgCAAAGGTCAAGGA
	TBP_rev	ENSG00000112592	TATA box binding protein	CCCCGCCTTTTATAC	AAGA	Reverse	caaaCCCCGCCTTTTATACcaaagaGTCTTCaaaGAAGACacactcAAGA	gagtTCTTgagtgtGTCTTCtttGAAGACtctttgGTATAAAAGGCGGGG	caaaCCCCGCCTTTTATACcaaagGAGACCaaaGGTCTCcactcAAGA	gagtTCTTgagtgGAGACCtttGGTCTCctttgGTATAAAAGGCGGGG
	FOXA2_rev	ENSG00000125798	forkhead box A2	CCTAAGTAAACA	GTGA	Reverse	caaaCCTAAGTAAACAcaaagaGTCTTCaaaGAAGACacactcGTGA	gagtTCACgagtgtGTCTTCtttGAAGACtctttgTGTTTACTTAGG	caaaCCTAAGTAAACAcaaagGAGACCaaaGGTCTCcactcGTGA	gagtTCACgagtgGAGACCtttGGTCTCctttgTGTTTACTTAGG
	FOXD1_rev	ENSG00000251493	forkhead box D1	ATGTTTAC	AAGC	Reverse	caaaATGTTTACcaaagaGTCTTCaaaGAAGACacactcAAGC	gagtGCTTgagtgtGTCTTCtttGAAGACtctttgGTAAACAT	caaaATGTTTACcaaagGAGACCaaaGGTCTCcactcAAGC	gagtGCTTgagtgGAGACCtttGGTCTCctttgGTAAACAT
	FOXF2_rev	ENSG00000137273	forkhead box F2	ATTGTTTACGTTTG	GGTT	Reverse	caaaATTGTTTACGTTTGcaaagaGTCTTCaaaGAAGACacactcGGTT	gagtAACCgagtgtGTCTTCtttGAAGACtctttgCAAACGTAAACAAT	caaaATTGTTTACGTTTGcaaagGAGACCaaaGGTCTCcactcGGTT	gagtAACCgagtgGAGACCtttGGTCTCctttgCAAACGTAAACAAT
	FOXA1_rev	ENSG00000129514	forkhead box A1	CAAAGTAAACATGGA	GGGT	Reverse	caaaCAAAGTAAACATGGAcaaagaGTCTTCaaaGAAGACacactcGGGT	gagtACCCgagtgtGTCTTCtttGAAGACtctttgTCCATGTTTACTTTG	caaaCAAAGTAAACATGGAcaaagGAGACCaaaGGTCTCcactcGGGT	gagtACCCgagtgGAGACCtttGGTCTCctttgTCCATGTTTACTTTG
	PLAG1_rev	ENSG00000181690	pleiomorphic adenoma gene 1	CCCCCTTGGGCCCC	TCAA	Reverse	caaaCCCCCTTGGGCCCCcaaagaGTCTTCaaaGAAGACacactcTCAA	gagtTTGAgagtgtGTCTTCtttGAAGACtctttgGGGGCCCAAGGGGG	caaaCCCCCTTGGGCCCCcaaagGAGACCaaaGGTCTCcactcTCAA	gagtTTGAgagtgGAGACCtttGGTCTCctttgGGGGCCCAAGGGGG
	GABPA_rev	ENSG00000154727	GA binding protein transcription factor, alpha subunit 60kDa	GCCACTTCCGG	GGCA	Reverse	caaaGCCACTTCCGGcaaagaGTCTTCaaaGAAGACacactcGGCA	gagtTGCCgagtgtGTCTTCtttGAAGACtctttgCCGGAAGTGGC	caaaGCCACTTCCGGcaaagGAGACCaaaGGTCTCcactcGGCA	gagtTGCCgagtgGAGACCtttGGTCTCctttgCCGGAAGTGGC
	MYB_rev	ENSG00000118513	v-myb myeloblastosis viral oncogene homolog (avian)	GACAGTTGGT	CAAT	Reverse	caaaGACAGTTGGTcaaagaGTCTTCaaaGAAGACacactcCAAT	gagtATTGgagtgtGTCTTCtttGAAGACtctttgACCAACTGTC	caaaGACAGTTGGTcaaagGAGACCaaaGGTCTCcactcCAAT	gagtATTGgagtgGAGACCtttGGTCTCctttgACCAACTGTC
	NFE2L2_rev	ENSG00000116044	nuclear factor (erythroid-derived 2)-like 2	TGCTGAGTCAT	GACA	Reverse	caaaTGCTGAGTCATcaaagaGTCTTCaaaGAAGACacactcGACA	gagtTGTCgagtgtGTCTTCtttGAAGACtctttgATGACTCAGCA	caaaTGCTGAGTCATcaaagGAGACCaaaGGTCTCcactcGACA	gagtTGTCgagtgGAGACCtttGGTCTCctttgATGACTCAGCA
	CREB1_rev	ENSG00000118260	cAMP responsive element binding protein 1	GGTGACGTCACC	CTCG	Reverse	caaaGGTGACGTCACCcaaagaGTCTTCaaaGAAGACacactcCTCG	gagtCGAGgagtgtGTCTTCtttGAAGACtctttgGGTGACGTCACC	caaaGGTGACGTCACCcaaagGAGACCaaaGGTCTCcactcCTCG	gagtCGAGgagtgGAGACCtttGGTCTCctttgGGTGACGTCACC
	EGR1_rev	ENSG00000120738	Early Growth Response 1	AATGCGTGGGCGTA	ATTT	Reverse	caaaAATGCGTGGGCGTAcaaagaGTCTTCaaaGAAGACacactcATTT	gagtAAATgagtgtGTCTTCtttGAAGACtctttgTACGCCCACGCATT	caaaAATGCGTGGGCGTAcaaagGAGACCaaaGGTCTCcactcATTT	gagtAAATgagtgGAGACCtttGGTCTCctttgTACGCCCACGCATT
	E2F1_rev	ENSG00000101412	E2F1	TTTGGCGCCAAA	CCTT	Reverse	caaaTTTGGCGCCAAAcaaagaGTCTTCaaaGAAGACacactcCCTT	gagtAAGGgagtgtGTCTTCtttGAAGACtctttgTTTGGCGCCAAA	caaaTTTGGCGCCAAAcaaagGAGACCaaaGGTCTCcactcCCTT	gagtAAGGgagtgGAGACCtttGGTCTCctttgTTTGGCGCCAAA
	NRF1_rev	ENSG00000106459	Nuclear respiratory factor 1	TGCGCGTGCGCA	TTGG	Reverse	caaaTGCGCGTGCGCAcaaagaGTCTTCaaaGAAGACacactcTTGG	gagtCCAAgagtgtGTCTTCtttGAAGACtctttgTGCGCACGCGCA	caaaTGCGCGTGCGCAcaaagGAGACCaaaGGTCTCcactcTTGG	gagtCCAAgagtgGAGACCtttGGTCTCctttgTGCGCACGCGCA

Supplementary Table 2: Identity of the top 3 promoters from each library used in studies.

Library	Promoter Name	Promoter Size (with SCP2)	BC1	BC2	BC3	BC4	BC5	BC6	BC7	BC8	Promoter Sequence (with SCP2)	Promoter Sequence with double enhancer, if applicable
1	Lib1-1	193	JUN_rev	NFE2L2_rev	EGR1	KLF6_rev	NFYA	SP1_rev	CEBPB		ATGACATCATCTTCAAATGCTGAGTCATCAAACCCCCGCCCCCGCCCAAATGGGCGTGGCCAAACTCAGCCAATCAGCGCAAAACCCCGCCCCCAAATATTGCACAATcaaaAGGTCTATATAAGCAGAGCTCGTTTAGTGAACCGTCAGATCGCCTGGAGACGTCGAGCCGAGTGGTTGTGCCTCCATAGAA	
1	Lib1-2	193	NR1H2_rev	POU2F1	TCF12_rev	ATF4_rev	FOS_rev	JUN_rev	ATF4_rev		GTTGACCTTTGACCTTTCAAAAATATGCAAATAACAAAGCACGTGCAAAATTGCATCATCCCAAAATGAGTCACACAAAATGACATCATCTTCAAAATTGCATCATCCcaaaAGGTCTATATAAGCAGAGCTCGTTTAGTGAACCGTCAGATCGCCTGGAGACGTCGAGCCGAGTGGTTGTGCCTCCATAGAA	GTTGACCTTTGACCTTTCAAAAATATGCAAATAACAAAGCACGTGCAAAATTGCATCATCCCAAAATGAGTCACACAAAATGACATCATCTTCAAAATTGCATCATCCGAACGTTGACCTTTGACCTTTCAAAAATATGCAAATAACAAAGCACGTGCAAAATTGCATCATCCCAAAATGAGTCACACAAAATGACATCATCTTCAAAATTGCATCATCCcaaaAGGTCTATATAAGCAGAGCTCGTTTAGTGAACCGTCAGATCGCCTGGAGACGTCGAGCCGAGTGGTTGTGCCTCCATAGAA
1	Lib1-3	201	NFE2L2	CREB1_rev	CEBPB	FOS_rev	SP1_rev	ATF4_rev	JUN_rev	POU2F1_rev	ATGACTCAGCACAAATGACGTCACAAATATTGCACAATCAAAATGAGTCACACAAAACCCCGCCCCCAAAATTGCATCATCCCAAAATGACATCATCTTCAAATTATTTGCATATTcaaaAGGTCTATATAAGCAGAGCTCGTTTAGTGAACCGTCAGATCGCCTGGAGACGTCGAGCCGAGTGGTTGTGCCTCCATAGAA	
2	Lib2-1	213	FOXA1_rev	FOXF2_rev	FOXD1_rev	NR2F1_rev	GABPA	EGR1	GABPA		CAAAGTAAACATGGACAAAATTGTTTACGTTTGCAAAATGTTTACCAAATCCTTGACCTTTGCAAACCGGAAGTGGCCAAATACGCCCACGCATTCAAATACGCCCACGCATTCAAACCGGAAGTGGCcaaaAGGTCTATATAAGCAGAGCTCGTTTAGTGAACCGTCAGATCGCCTGGAGACGTCGAGCCGAGTGGTTGTGCCTCCATAGAA	
2	Lib2-2	218	EGR1	HNF1A_rev	NR2F1_rev	E2F1_rev	CREB1_rev	NFE2L2_rev	FOXF2	TBP	TACGCCCACGCATTCAAAAGTTAATCATTAACTCAAATGCGCGTGCGCACAAATTTGGCGCCAAACAAAGGTGACGTCACCCAAATGCTGAGTCATCAAACAAACGTAAACAATCAAAGTATAAAAGGCGGGGcaaaAGGTCTATATAAGCAGAGCTCGTTTAGTGAACCGTCAGATCGCCTGGAGACGTCGAGCCGAGTGGTTGTGCCTCCATAGAA	TACGCCCACGCATTCAAAAGTTAATCATTAACTCAAATGCGCGTGCGCACAAATTTGGCGCCAAACAAAGGTGACGTCACCCAAATGCTGAGTCATCAAACAAACGTAAACAATCAAAGTATAAAAGGCGGGGGAACTACGCCCACGCATTCAAAAGTTAATCATTAACTCAAATGCGCGTGCGCACAAATTTGGCGCCAAACAAAGGTGACGTCACCCAAATGCTGAGTCATCAAACAAACGTAAACAATCAAAGTATAAAAGGCGGGGcaaaAGGTCTATATAAGCAGAGCTCGTTTAGTGAACCGTCAGATCGCCTGGAGACGTCGAGCCGAGTGGTTGTGCCTCCATAGAA
2	Lib2-3	175	NR2F1_rev	NFE2L2	NFE2L2	NR2F1_rev	NFE2L2	NFE2L2_rev			TCCTTGACCTTTGCAAAATGACTCAGCACAAAATGACTCAGCACAAATCCTTGACCTTTGCAAAATGACTCAGCACAAATGCTGAGTCATcaaaAGGTCTATATAAGCAGAGCTCGTTTAGTGAACCGTCAGATCGCCTGGAGACGTCGAGCCGAGTGGTTGTGCCTCCATAGAA	

Supplementary Table 3: Sequences of constitutive promoters used in studies

Promoter Name	Length	Sequence
CAG	1664	actagttattaatagtaatcaattacggggtcattagttcatagcccatatatggagttccgcgttacataacttacggtaaatggcccgcctggctgaccgcccaacgacccccgcccattgacgtc<br>aataatgacgtatgttcccatagtaacgccaatagggactttccattgacgtcaatgggtggagtatttacggtaaactgcccacttggcagtacatcaagtgtatcatatgccaagtacgccccctat<br>tgacgtcaatgacggtaaatggcccgcctggcattatgcccagtacatgaccttatgggactttcctacttggcagtacatctacgtattagtcatcgctattaccatggtcgaggtgagccccacgttc<br>tgcttcactctccccatctcccccccctccccacccccaattttgtatttatttattttttaattattttgtgcagcgatgggggcgggggggggggggggcgcgcgccaggcggggcggggcggggcga<br>ggggcggggcggggcgaggcggagaggtgcggcggcagccaatcagagcggcgcgctccgaaagtttccttttatggcgaggcggcggcggcggcggccctataaaaagcgaagcgcgcggcgggcg<br>ggagtcgctgcgacgctgccttcgccccgtgccccgctccgccgccgcctcgcgccgcccgccccggctctgactgaccgcgttactcccacaggtgagcgggcgggacggcccttctcctccgg<br>gctgtaattagcgcttggtttaatgacggcttgtttcttttctgtggctgcgtgaaagccttgaggggctccgggagggccctttgtgcggggggagcggctcggggggtgcgtgcgtgtgtgtgtgcgtggggag<br>cgccgcgtgcggctccgcgctgcccggcggctgtgagcgctgcgggcgcggcgcggggctttgtgcgctccgcagtgtgcgcgaggggagcgcggccgggggcggtgccccgcggtgcggggggggctg<br>cgaggggaacaaaggctgcgtgcggggtgtgtgcgtgggggggtgagcagggggtgtgggcgcgtcggtcgggctgcaaccccccctgcacccccctccccgagttgctgagcacggcccggcttcgg<br>gtgcggggctccgtacggggcgtggcgcggggctcgccgtgccgggcggggggtggcggcaggtgggggtgccgggcggggcggggccgcctcgggccggggagggctcgggggaggggcgcggcggcc<br>cccggagcgccggcggctgtcgaggcgcggcgagccgcagccattgccttttatggtaatcgtgcgagagggcgcagggacttcctttgtcccaaatctgtgcggagccgaaatctgggaggcgccgc<br>cgcaccccctctagcgggcgcggggcgaagcggtgcggcgccggcaggaaggaaatgggcggggagggccttcgtgcgtcgccgcgccgccgtccccttctccctctccagcctcggggctgtccg<br>cggggggacggctgccttcgggggggacggggcagggcggggttcggcttctggcgtgtgaccggcggctctagagcctctgctaaccatgttcatgccttcttctttttcctacag
Mini-CMV	173	ACTCACGGGGATTTCCAAGTCTCCACCCCATTGACGTCAATGGGAGTTTGTTTTGGCACCAAAATCAACGGGACTTTCCAAAATGTCGTAATAACCCCGC<br>CCCGTTGACGCAAATGGGCGGTAGGCGTGTACGGTGGGAGGTCTATATAAGCAGAGCTCGTTTAGTGAACCGT
Full-CMV	811	Acattgattattgactagttattaatagtaatcaattacggggtcattagttcatagcccatatatggagttccgcgttacataacttacggtaaatggcccgcctggctgaccgcccaacgacccccg<br>cccattgacgtcaataatgacgtatgttcccatagtaacgccaatagggactttccattgacgtcaatgggtggagtatttacggtaaactgcccacttggcagtacatcaagtgtatcatatgccaag<br>tccgccccctattgacgtcaatgacggtaaatggcccgcctggcattatgcccagtacatgaccttacgggactttcctacttggcagtacatctacgtattagtcatcgctattaccatggtgatgcg<br>gttttggcagtacaccaatgggcgtggatagcggtttgactcacggggatttccaagtctccaccccattgacgtcaatgggagtttgttttggcaccaaaatcaacgggactttccaaaatgtcgtaat<br>aaccccgccccgttgacgcaaatgggcggtaggcgtgtacggtgggaggtctatataagcagaggtcgtttagtgaaccgtcagatcactagtagctttattgcggtagtttatcacagttaaattgcta<br>acgcagtcagtgctcgactgatcacaggtaagtatcaaggttacaagacaggtttaaggaggccaatagaaactgggcttgtcgagacagagaagattcttgcgtttctgataggcacctattggtctt<br>actgacatccactttgcctttctctccacagggcg
HLP	252	tgtttgctgcttgcaatgtttgcccattttagggtggacacaggacgctgtggtttctgagccagggggcgactcagatcccagccagtggacttagcccctgtttgctcctccgataactggggtgacctt<br>ggttaatattcaccagcagcctcccccgttgcccctctggatccactgcttaaatacggacgaggacagggccctgtctcctcagcttcaggcaccaccactgacctgggacagtgaatc

Supplementary Table 4: Introns tested in top promoters

Name	Size	Sequence
Full-CMV Intron	228	cgtttagtgaaccgtcagatcactagtagctttattgcggtagtttatcacagttaaattgctaacgcagtcagtgctcgactgatcacaggtaagtatcaaggttacaagacaggtttaaggaggccaatagaaactgggcttgtcgagacagagaagattcttgcgtttctgataggcacctattggtcttactgacatccactttgcctttctctccacagggcg
Mini-CMV Intron	133	gtaagtatcaaggttacaagacaggtttaaggaggccaatagaaactgggcttgtcgagacagagaagattcttgcgtttctgataggcacctattggtcttactgacatccactttgcctttctctccacag
SV40 Intron	93	ctctaaggtaaatataaaatttttaagtgtataatgtgttaaactactgattctaattgtttctctcttttagattccaacctttggaactga

Supplementary Table 5: Modifications to BDDFVIII gene used in studies

Original BDDFVIII sequence	Length	X5 Modifications	Resource
atgcaaatagagctctccacctgcttctttctgtgccttttgcgattctgctttagtgccaccagaagatactacctgggtgcagtggaactgtcatgggactatatgcaaagtgatctcggtgagctgcctgtggacgcaagatttcctcctagagtgccaaaatcttttccattcaacacctcagtcgtgtacaaaaagactctgtttgtagaattcacggatcaccttttcaacatcgctaagccaaggccaccctggatgggtctgctaggtcctaccatccaggctgaggtttatgatacagtggtcattacacttaagaacatggcttcccatcctgtcagtcttcatgctgttggtgtatcctactggaaagcttctgagggagctgaatatgatgatcagaccagtcaaagggagaaagaagatgataaagtcttccctggtggaagccatacatatgtctggcaggtcctgaaagagaatggtccaatggcctctgacccactgtgccttacctactcatatctttctcatgtggacctggtaaaagacttgaattcaggcctcattggagccctactagtatgtagagaagggagtctggccaaggaaaagacacagaccttgcacaaatttatactactttttgctgtatttgatgaagggaaaagttggcactcagaaacaaagaactccttgatgcaggatagggatgctgcatctgctcgggcctggcctaaaatgcacacagtcaatggttatgtaaacaggtctctgccaggtctgattggatgccacaggaaatcagtctattggcatgtgattggaatgggcaccactcctgaagtgcactcaatattcctcgaaggtcacacatttcttgtgaggaaccatcgccaggcgtccttggaaatctcgccaataactttccttactgctcaaacactcttgatggaccttggacagtttctactgttttgtcatatctcttcccaccaacatgatggcatggaagcttatgtcaaagtagacagctgtccagaggaaccccaactacgaatgaaaaataatgaagaagcggaagactatgatgatgatcttactgattctgaaatggatgtggtcaggtttgatgatgacaactctccttcctttatccaaattcgctcagttgccaagaagcatcctaaaacttgggtacattacattgctgctgaagaggaggactgggactatgctcccttagtcctcgcccccgatgacagaagttataaaagtcaatatttgaacaatggccctcagcggattggtaggaagtacaaaaaagtccgatttatggcatacacagatgaaacctttaagactcgtgaagctattcagcatgaatcaggaatcttgggacctttactttatggggaagttggagacacactgttgattatatttaagaatcaagcaagcagaccatataacatctaccctcacggaatcactgatgtccgtcctttgtattcaaggagattaccaaaaggtgtaaaacatttgaaggattttccaattctgccaggagaaatattcaaatataaatggacagtgactgtagaagatgggccaactaaatcagatcctcggtgcctgacccgctattactctagtttcgttaatatggagagagatctagcttcaggactcattggccctctcctcatctgctacaaagaatctgtagatcaaagaggaaaccagataatgtcagacaagaggaatgtcatcctgttttctgtatttgatgagaaccgaagctggtacctcacagagaatatacaacgctttctccccaatccagctggagtgcagcttgaggatccagagttccaagcctccaacatcatgcacagcatcaatggctatgtttttgatagtttgcagttgtcagtttgtttgcatgaggtggcatactggtacattctaagcattggagcacagactgacttcctttctgtcttcttctctggatataccttcaaacacaaaatggtctatgaagacacactcaccctattcccattctcaggagaaactgtcttcatgtcgatggaaaacccaggtctatggattctggggtgccacaactcagactttcggaacagaggcatgaccgccttactgaaggtttctagttgtgacaagaacactggtgattattacgaggacagttatgaagatatttcagcatacttgctgagtaaaaacaatgccattgaaccaagaagcttctctcaaaacccaccagtcttgaaacgccatcaacgggaaataactcgtactactcttcagtcagatcaagaggaaattgactatgatgataccatatcagttgaaatgaagaaggaagattttgacatttatgatgaggatgaaaatcagagcccccgcagctttcaaaagaaaacacgacactattttattgctgcagtggagaggctctgggattatgggatgagtagctccccacatgttctaagaaacagggctcagagtggcagtgtccctcagttcaagaaagttgttttccaggaatttactgatggctcctttactcagcccttataccgtggagaactaaatgaacatttgggactcctggggccatatataagagcagaagttgaagataatatcatggtaactttcagaaatcaggcctctcgtccctattccttctattctagccttatttcttatgaggaagatcagaggcaaggagcagaacctagaaaaaactttgtcaagcctaatgaaaccaaaacttacttttggaaagtgcaacatcatatggcacccactaaagatgagtttgactgcaaagcctgggcttatttctctgatgttgacctggaaaaagatgtgcactcaggcctgattggaccccttctggtctgccacactaacacactgaaccctgctcatgggagacaagtgacagtacaggaatttgctctgtttttcaccatctttgatgagaccaaaagctggtacttcactgaaaatatggaaagaaactgcagggctccctgcaatatccagatggaagatcccacttttaaagagaattatcgcttccatgcaatcaatggctaca	4374	V86I, S108A, K132G, T147M, P152L	Cao, W. *et al.* Molecular Therapy: Methods & Clinical Development (2020).

Minimal polyA sequence	Length	Resource
aataaaagatctttattttcattagatctgtgtgttggttttttgtgtg	49	Choi, J.-H. *et al.* Molecular Brain (2014).
